# The formation of a glial scar does not prohibit remyelination in an animal model of multiple sclerosis

**DOI:** 10.1002/glia.23556

**Published:** 2018-11-28

**Authors:** Michaela Tanja Haindl, Ulrike Köck, Milena Zeitelhofer‐Adzemovic, Franz Fazekas, Sonja Hochmeister

**Affiliations:** ^1^ Department of Neurology Medical University of Graz Graz Austria; ^2^ Center for Brain Research Medical University of Vienna Vienna Austria; ^3^ Department of Clinical Neuroscience, Center for Molecular Medicine Karolinska Institutet Stockholm Sweden

**Keywords:** astrocytes, glial scar, multiple sclerosis, oligodendrocyte precursor cells, remyelination, shadow plaque

## Abstract

The role of astrocytes in the pathophysiology of multiple sclerosis (MS) is discussed controversially. Especially the formation of the glial scar is often believed to act as a barrier for remyelination. At the same time, astrocytes are known to produce factors that influence oligodendrocyte precursor cell (OPC) survival. To explore these mechanisms, we investigated the astrocytic reaction in an animal model induced by immunization with myelin oligodendrocyte glycoprotein (MOG) in Dark Agouti (DA) rats, which mimics most of the histological features of MS. We correlated the astroglial reaction by immunohistochemistry (IHC) for glial fibrillary acidic protein (GFAP) to the remyelination capacity by in situ hybridization for mRNA of proteolipid protein (PLP), indicative of OPCs, over the full course of the disease. PLP mRNA peaked in early remyelinating lesions while the amount of GFAP positive astrocytes was highest in remyelinated lesions. In shadow plaques, we found at the same time all features of a glial scar and numbers of OPCs and mature oligodendrocytes, which were nearly equal to that in unaffected white matter areas. To assess the plaque environment, we furthermore quantitatively analyzed factors expressed by astrocytes previously suggested to influence remyelination. From our data, we conclude that remyelination occurs despite an abundant glial reaction in this animal model. The different patterns of astrocytic factors and the occurrence of different astrocytic phenotypes during lesion evolution furthermore indicate a finely regulated, balanced astrocytic involvement leading to successful repair.

## 1 INTRODUCTION

Astrocytes are the most abundant cell type in the mammalian brain, outnumbering neurons by over fivefold (Sofroniew & Vinters, 2010). In the healthy central nervous system (CNS), astrocytes play important roles in supporting synaptic transmission and providing functional support of neurons by maintaining local ion and pH homeostasis, storing CNS glycogen, clearing neuronal waste, and providing maintenance and modulation of the blood‐brain barrier (Nair, Frederick, & Miller, 2008). Astrocytes react to all sorts of insults to the CNS in a process referred to as reactive astrogliosis, which is often seen as pathological hallmark of diseased CNS tissue. These changes range from rather mild reversible alterations with cellular hypertrophy without overlap of astrocyte processes to long‐lasting scar formation. The glial scar is associated with a rearrangement of tissue structure, astrocyte proliferation, and a pronounced overlap of astrocyte processes, which result in a disruption of individual astrocyte domains (Sofroniew & Vinters, 2010). Pathologically the hallmark of astrogliosis and activated astrocytes is an upregulation of glial fibrillary acidic protein (GFAP), which belongs to the family of the intermediate filament proteins and is commonly used as a marker for astrocytes in histology. Mild to moderate astrogliosis is believed to protect CNS cells and tissue by uptake of potentially excitotoxic glutamate, protects from oxidative stress, facilitates the blood–brain barrier repair and limits the spread of inflammatory cells or infectious agents from areas of damage or disease into healthy CNS tissue (Iglesias, Morales, & Barreto, 2017; Ludwin, Rao, Moore, & Antel, 2016; Nair et al., 2008; Sofroniew & Vinters, 2010). In contrast, the formation of a compact glial scar is believed to act as an inhibitor of regeneration because of its mechanical rigidity, especially in the scenario of multiple sclerosis (MS) (Brambilla et al., 2014; Ghatak, 1992). This has even led to experimental approaches which aimed at eliminating reactive astrocytes or prevent them from becoming fully reactive to foster regeneration (Correale & Farez, 2015). Contrariwise astrocytes were shown to produce several factors, which facilitate repair processes upon activation.

The most common model for studying MS is experimental autoimmune encephalomyelitis (EAE) induced in rodents by active immunization with myelin oligodendrocyte glycoprotein (MOG) because it mimics many of the pathophysiological mechanisms and histopathological features of this disease. Histologically it allows to study the full course of lesion evolution from active demyelination up to successful remyelination, the shadow plaque (SP) (Gold, Hartung, & Toyka, 2000; Weissert et al., 1998). The aim of our study was to investigate the astrocytic reaction at different stages of lesion evolution and the correlation between the extent of astrogliosis and remyelination in MOG EAE. To gain more information about the role of astrocytes in remyelination, we furthermore investigated the distribution of a selected set of astrocyte‐derived factors known for their involvement during lesion evolution. We hypothesized that a complex arrangement of these factors is needed at specific time points to result in successful remyelination. Furthermore, we followed the two astrocytic phenotypes A1 and A2 during lesion evolution where the A1 phenotype has been suggested to prohibit remyelination, whereas A2 appears to be the remyelination promoting phenotype (Liddelow et al., 2017). We discuss the expression pattern seen in our experimental sample of successful remyelination as opposed to the available data in human MS brain specimen, where often remyelination is not successfully accomplished. Understanding the role of astrocytes in remyelination could lead to a better knowledge of repair mechanisms and how they could be influenced.

## 2 MATERIALS AND METHODS

### 2.1 Used material, selection of lesions, and definition of lesional staging

The study was performed on archival material from previous EAE experiments in female DA/OlaHsd rats 8–10 weeks actively immunized with recombinant rat MOG (rrMOG), corresponding to the N‐terminal sequence of rat MOG (aa1–125) collected within the archives of the neuroimmunology research laboratory between 1998 until present (Hochmeister et al., 2012; Seifert, Bauer, Weissert, Fazekas, & Storch, 2007; Storch et al., 2006). Detailed information on clinical course of the disease is given in the respective references (Storch et al., 1998; Weissert et al., 1998, 2003). For the purpose of our investigation, we selected material from animals with clinical symptoms and large confluent lesions which led to a total of 45 animals. Lesions were classified as active lesion (A), inactive to early remyelinating lesion (IA/ER), early to late remyelinating lesion (ER/LR), late remyelinating lesion to SP (LR/SP) and SP following previously suggested criteria (Kornek et al., 2000; Lucchinetti et al., 1999). In short, actively demyelinating lesions are defined by the presence of macrophages containing luxol fast blue (LFB) positive myelin degradation products, which are immunoreactive for myelin proteins. In early active lesions, these degradation products are immunoreactive for all myelin proteins including MOG, whereas in late active lesions the degradation products are immunoreactive for the major myelin proteins, myelin binding protein (MBP) and proteolipid protein (PLP) but not for MOG. We validated myelin degradation products within macrophages with immunofluorescent (IF) double staining against CD68 (ED1, a marker for macrophages) and MOG. In inactive demyelinated lesions, macrophages may be loaded with PAS‐positive granular or sudanophilic lipids but do not contain myelin degradation products (Lucchinetti, Brück, Rodriguez, & Lassmann, 1996). Early remyelinating lesions are similar to inactive demyelinated plaques but contain myelin fibers positive for certain myelin proteins. Late remyelinating lesions (classical SPs) show a reduced staining intensity (myelin pallor) in conventional myelin staining (LFB) compared to the periplaque white matter due to thin, newly formed myelin sheaths. Areas without any histopathological changes, that is normal appearing white matter (NAWM), served as control regions (Zeis, Graumann, Reynolds, & Schaeren‐Wiemers, 2008).

### 2.2 Tissue processing, immunohistochemistry and immunofluorescence

Serial paraffin sections from 1.5 to 2 μm were prepared (for in situ hybridization under RNase‐free conditions) and dewaxed in Xylene (Roth, Karlsruhe, Germany). Slides were stained for hematoxylin & eosin (H & E) and LFB to obtain a first overview on tissue inflammation and demyelination.

For immunohistochemistry (IHC), slides were dewaxed, rehydrated, and incubated in a methanol solution containing H_2_O_2_ (Merck, Darmstadt, Germany) to be able to block the endogenous peroxidase (Bauer et al., 2002). Antigen retrieval was performed using EDTA (Sigma, St. Louis, Missouri, USA) at pH 5.2 in a steamer for 1 hr. After a cooling step, slides were washed and blocked for 20 min with 10% fetal calf serum (FCS; Sigma) in DAKO REAL Antibody diluent (Dako; Santa Clara, CA). The blocking solution was discarded and the primary antibody was applied at a dilution of 1:100 in FCS/DAKO and incubated over night at 4 °C. After three washing steps with PBS, the secondary biotinylated antibody (GE Healthcare, Buckinghamshire, Great Britain) was applied at a dilution of 1:200 in FCS/DAKO and incubated at room temperature for 1 hr. Coated with peroxidase labeled avidin (Sigma) diluted 1:100 in FCS/DAKO slides were incubated at room temperature for 1 hr. Visualization of the primary antibody was then performed with 3,3′‐diaminobenzidine (DAB; Sigma) and counterstaining with hematoxylin (Gatt‐Koller, Absam, Austria). Slides were dehydrated in ascending ethanol concentration and covered with Shandon Consul‐Mount (Thermo Scientific, Waltham, Massachusetts, USA) and a cover slip.

For IHC double staining, pretreatment procedures were performed as described earlier. GFAP and one astrocytic factor, respectively, were incubated simultaneously in FCS/DAKO at 4 °C overnight on selected lesions. For the detection of primary antibodies, a polymer‐based detection system (ImmPress; Vector laboratories, Burlingame, CA) was used according to the manufacturer's instructions. Visualization of anti‐mouse ImmPress reagent was performed with DAB (Sigma) and visualization of anti‐rabbit ImmPress or anti‐goat ImmPress reagent, respectively, were obtained with VIP Peroxidase substrate kit (Vector Laboratories). Afterward, slides were dehydrated and covered with Shandon Consul‐Mount (Thermo Scientific) and a coverslip. Further information about all used antibodies can be found in the Supporting Information Table S1.

For immunofluorescence (IF), double staining pretreatment and primary antibody incubation were the same as described earlier. For the detection of the two different antibodies, we used VectaFluor DyLight 594 anti‐mouse and VectaFluor DyLight 488 anti‐rabbit (Vector Laboratories). Slides were not dehydrated but covered aqueous with Vectashield HardSet Mounting Medium (Vector Laboratories).

### 2.3 In situ hybridization/IHC double staining

For in situ hybridization (ISH), all steps were conducted with diethylpyrocarbonate (DEPC; Sigma) water to ensure RNase‐free conditions. The paraffin‐embedded tissue sections were dewaxed in Xylene (Roth) and rehydrated. To fix the intersections, slides were incubated in 4% PFA (Merck) for 20 min. After washing steps in tris‐buffered‐saline (TBS; Merck) slides were incubated in 0.2 M HCl (Sigma) for 10 min. Pretreatment was performed with 20 μg/ml proteinase K solution (Sigma) for 20 min at 37 °C. After stopping the enzyme activity in TBS at 4 °C, slides were incubated in 0.5% acetic anhydride (Sigma) under stirring for 10 min to block the endogenous alkaline phosphatase reaction. Slides were dehydrated and incubated in a wet chamber at 55 °C for 30 min. The full length probes (kindly provided by the institute of brain research Vienna), were applied, coverslipped, and incubated for exactly 4 min on a 95 °C heating plate. Hybridization occurred over night at 65 °C. On the following day, slides were washed and incubated in Boehringer blocking reagents (Roche, IN) with 10% FCS for 15 min. Anti‐digoxigenin antibodies conjugated with alkaline phosphatase (Roche) were applied at a dilution of 1:500 in blocking reagent with 10% FCS for 1 hr. The sections were developed with NBT/BCIP (Roche) at 4 °C under microscopic controls.

For the immunohistochemical double staining, mouse PLP primary antibodies were applied at a dilution of 1:1000 in 10% FCS/TBS over night at 4 °C. After some washing steps, the biotinylated secondary mouse antibody (GE Healthcare, Buckinghamshire, Great Britain) was applied at a dilution of 1:200 in FCS/TBS 1 hr at room temperature. Then incubation with avidin‐alkaline phosphatase complex (Roche) was performed for 1 hr at room temperature. The slides were developed using a fast red solution (Sigma). Counterstaining was performed with hematoxylin and slides were coverslipped as described earlier.

### 2.4 Histopathology and quantitative evaluation

The number and types of lesions and the number of rats which were available for GFAP‐IHC, PLP‐ISH, and NOGO‐IHC are given in Table 1. Analysis was performed by a single investigator blinded for lesion types (MTH). The selected lesions were quantitatively assessed on a Zeiss Axioplan2 imaging light microscope under a 400× magnification. All specifically stained cells for the respective antigen were quantitatively assessed in one full square grid and results converted into corresponding cells per square millimeter.

**Table 1:** Number of quantified lesions for GFAP‐IHC, PLP‐ISH, and NOGO‐IHC

	NAWM	A	IA/ER	ER/LR	LR/SP	SP	Total	Rats
GFAP‐IHC	77	116	84	88	90	85	540	45
PLP‐ISH	61	70	76	79	85	79	450	45
NOGO‐IHC	20	20	20	20	20	20	120	15

In total, GFAP immunoreactivity (GFAP‐IHC) was assessed in 540 lesions, OPC density (PLP‐ISH) in 450 lesions, and occurrence of mature Oligodendrocytes (NOGO‐IHC) in 120 lesions by one blinded investigator (MTH).

As shown in Table 1, the number of assessed lesions in GFAP‐IHC and PLP‐ISH varies between the staining types due to tissue loss or unspecific results during the staining process. These staining problems appear among others due to the polymerization of GFAP during astrocytic reaction, masking the epitopes (Ludwin et al., 2016). For NOGO‐IHC, we used a selected set of lesions. We investigated the lesion evolution in brain (cerebellum) and spinal cord specimens. For quantitative evaluation and statistical analysis, we only used spinal cord lesions to ensure comparability; however, there is a close histopathological resemblance of the lesions in the spinal cord and in the brain.

We chose GFAP as marker for astrocytes because of its best countability. For quality control, we stained two additional markers for astrocytes, Nestin and Vimentin. In Supporting Information Figure S1a–i, the three different markers are shown on the same lesions side‐by‐side for comparison. We also performed double staining of GLT1 (Excitatory Amino Acid Transporter 2 EAAT2, rodent analog GLT1) and GFAP on ER/LR lesions (Supporting Information Figure S1j). As postulated by Yang et al., 2011, GLT1 is primarily distributed at the membrane of astroglial processes thereby making it difficult to clearly identify individual astrocytes by their immunostaining (Yang et al., 2011). As GFAP is an intermediate filament the whole cell body is stained quite well, which makes it more practicable during quantification.

As the lesion characterization serves as basis of all presented research, we validated our characterization with IF staining against macrophages (ED1) and myelin components (MOG) as most of the characterization depends on the presence or absence of myelin degradation products within macrophages in lesions (Supporting Information Figure S1k,l).

### 2.5 Quantitative evaluation of double‐labeled cells and correlation analysis

For IHC double staining experiments, we selected 20 representative lesions (selected out of 15 rats) from every lesion type and NAWM according to best GFAP staining results and tissue quality of the whole sample set. As there are many factors expressed by astrocytes known to intervene during remyelination, we selected only a small subset composed of semaphorins 3A and 3F (Sema3A, Sema3F), hyaluronan (detected via hyaluronan binding protein 2, HABP2), bone morphogenetic protein‐2 (BMP‐2), ciliary neurotrophic factor (CNTF), brain‐derived neurotrophic factor (BDNF), CXC‐motif‐chemokine 12 (CXCL‐12), fibronectin 1 (FN1), tenascin‐C (Tn‐C), insulin‐like growth factor (IGF), fibroblast growth factor‐2 (FGF‐2), and interleukin‐6 (Il‐6).

Double‐labeled cells of GFAP and the respective astrocyte‐derived factor were counted within the lesions using an ocular morphometric grid under a 400× magnification. The size of the grid at this magnification was 313 μm × 313 μm. The values were then transformed to cells per square millimeter. The quantification of all factors in each lesion type is given as percentage scale with all detected GFAP positive astrocytes representing 100% and the corresponding ratio as the detected double‐labeled cells in the same area. We furthermore performed a correlation analysis of astrocytic factors and the extent of remyelination (OPC density) to examine their association.

### 2.6 Evaluation of astrocytic phenotypes A1 and A2

To follow the two different astrocytic phenotypes A1 and A2 over the full course of lesion evolution, we chose the markers described by Liddelow et al., 2017; C3d complement for detecting A1 and S100 calcium‐binding protein A10 (S100A10) for A2 (Liddelow et al., 2017). We documented the results via selected microscopy pictures.

### 2.7 Statistical analysis

All statistical calculations and graphic illustrations were performed using IBM SPSS Statistics 23 and Microsoft Excel 2010. First, we tested for a normal distribution of the data by performing the Kolmogorov–Smirnov test with most of the variables being not normally distributed. Because of this and the ordinal scaled lesion evolution, we used the nonparametric Kruskal–Wallis test (*p* < .05) to assess statistical differences between groups. With this test, we could detect significant differences and therefore added an analysis using the Mann–Whitney *U* test for multiple comparisons with Bonferroni correction (a cutoff for the *p*‐value of .01 was considered significant).

In a correlation analysis, we correlated OPC density with the respective factor occurrence during lesion evolution (A ‐ SP). For this purpose, we selected the same lesions for OPC as selected for all analyzed factors and used the Spearman‐Rho nonparametric correlation with *p* < .01 considered as significant (two‐tailed).

## 3 RESULTS

### 3.1 Histopathological changes in rat brains and spinal cords

White matter lesions in the brains (usually located in the cerebellar white matter and occasionally in the white matter of the corpus callosum) and—more often—in the spinal cords of DA rats as typically caused by the MOG EAE model were identified and analyzed with respect to lesion types as described earlier (Table 1).

**Figure 1:**
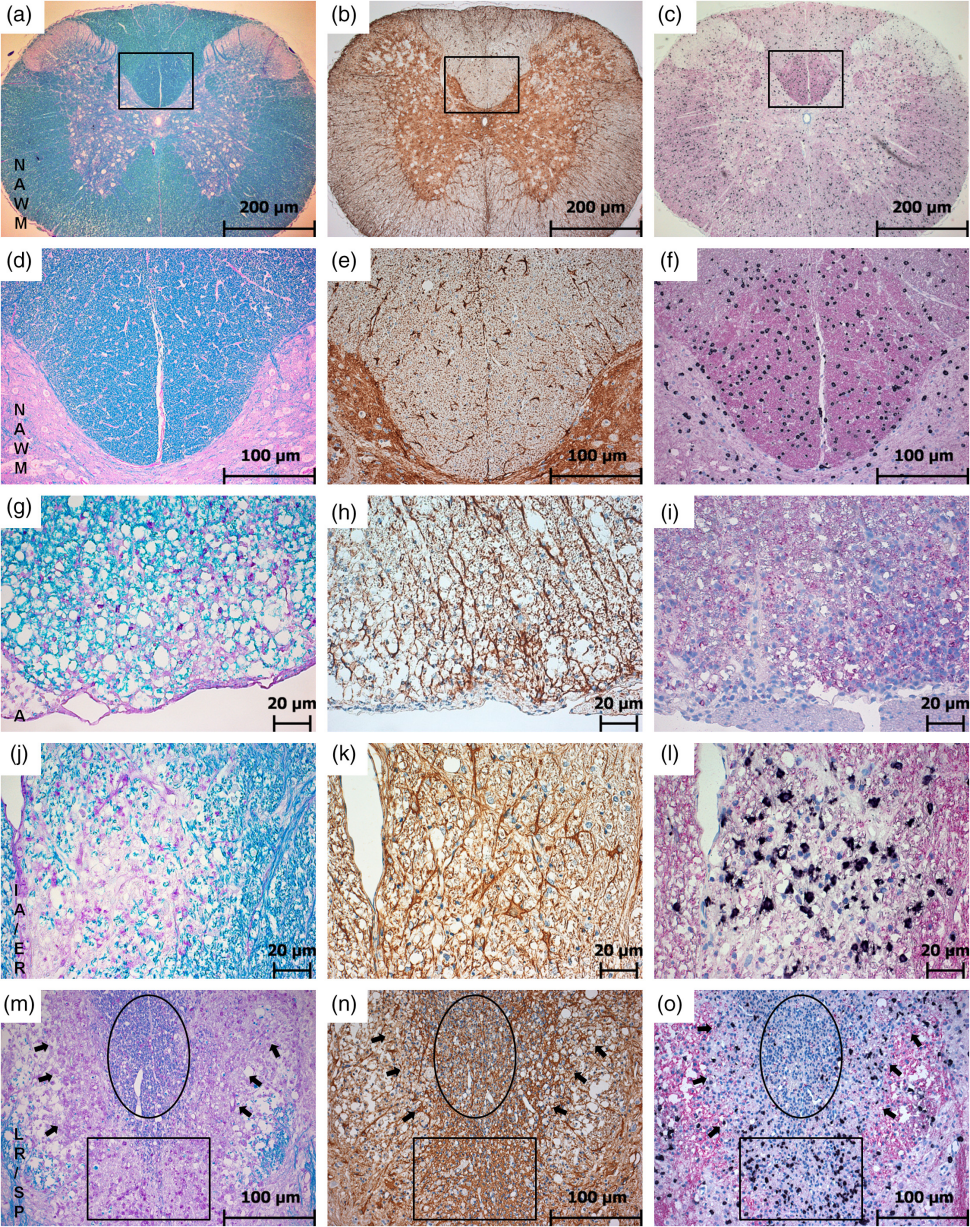
Lesion evolution in the rat spinal cord. This figure shows the results of LFB over the course of lesion evolution (first column: (a), (d), (g), (j), (m)). In this staining, lesion areas can be detected as an overview in pink and SPs are represented in pale blue due to thinner myelin sheaths after remyelination. GFAP‐IHC (second column: (b), (e), (h), (k), (n)) shows reactive astrocytes (dark brown) and is used to trace astrogliosis. PLP‐ISH (third column: (c), (f), (i), (l), (o)) shows OPC density via PLP mRNA ISH (black) and the corresponding protein (in pink) at the same time. The rectangles in the figures of the first line (a–c) indicate the areas in the second line at a higher magnification (d–f). The first and the second line show the NAWM with normal, dark blue myelin in LFB (a, d), only some active astrocytes in GFAP (b, e), and a normal distribution of OPCs with a strong PLP immunoreactivity (c, f). In active lesions pink areas represent the absent myelin in LFB; macrophages are carrying early (blue) myelin degradation products (g). Astrocytes are more activated and start to branch (h), OPCs are nearly absent and PLP loss is indicated by a pale pink area (j). IA/ER clearly set apart in pink in LFB and myelin degradation products are already digested and appear in pink as well (j). Astrocytes are getting even larger and more branched (k) and a massive accumulation of OPCs can be observed in the same area where PLP loss is detectable (l). Lesions tend to undergo smooth transition between different stages with different lesion types bordering (m–o). The circle marks the SP area in LFB (m), GFAP (n) and PLP (o). The rectangle indicates a part with ongoing ER and the arrows point at the border of LR (magnification: (a–c) 50×; (d–f) and (m–o) 200×; (g–l) 400×) [Color figure can be viewed at wileyonlinelibrary.com]

**Figure 2:**
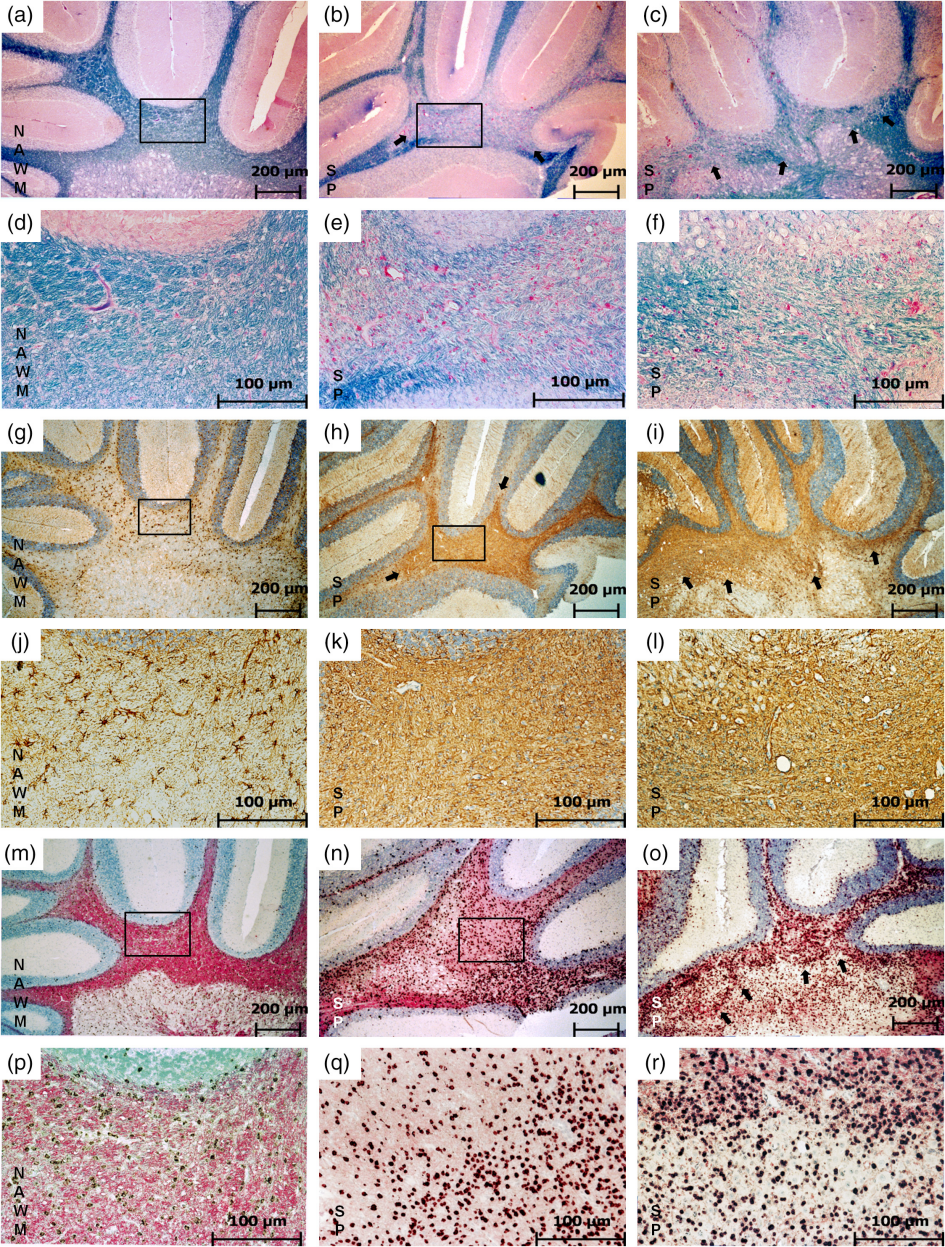
Shadow plaques in the rat brain. This figure shows a direct comparison of SP features and NAWM as found in the cerebellar white matter. Panels (a–f) represent LFB staining, (g–l) show GFAP‐IHC, (m–r) show PLP ISH. In NAWM (a, d) myelin appears in dark blue in LFB staining (a). The rectangle indicates the area represented in (d) at a higher magnification. The fully remyelinated SP appears in light blue due to thinner myelin sheaths after accomplished remyelination (b, c, e, f). The rectangle in (b) indicates the area shown in (e) and the arrows in (b) and (c) point at the SP border. Only some astrocytes appear GFAP positive in NAWM (g). The rectangle indicates the area represented in (j) at a higher magnification. In SP areas, astrocytes exhibit all features associated with a glial scar (h, i, k, l). The rectangle in (h) indicates the area represented in (k). The arrows in (h) and (i) point at the border of the glial scar. In NAWM, a normal occurrence of OPCs (black) and PLP (pink) can be observed (m). The rectangle indicates the area represented in (p) with a higher magnification. The distribution of OPCs in SP appears similar to their distribution in NAWM but with less PLP immunoreactivity in the background represented by a much paler pink background (n, o, q, r). The rectangle in (n) indicates the area shown in (q) and the arrows in (o) point to the SP border (magnification: (a–c), (g–i), (m–o) 50×; (d–f), (j–l), (p–r) 200×) [Color figure can be viewed at wileyonlinelibrary.com]

Adjacent slides of each lesion were subjected to IHC for GFAP to detect the astrocytic reaction and ISH for PLP mRNA to monitor remyelinating OPCs in spinal cords and brains. Figures 1 and 2 illustrate in detail the characteristics of the different lesion types as described in the following.

In the NAWM, intact myelin is shown as intense blue staining both in the spinal cord (Figure 1a,d) and in the brain (Figure 2a,d). The distribution of active astrocytes is rather sparse (Figure 1b,e), the single astrocytes are clearly separated from each other. A normal OPC distribution (represented by dark dots in the tissue) and a normal PLP immunoreactivity are shown in Figure 1c,f in the spinal cord and in Figure 2m,p in the brain.

Active (A) lesions appear pink in LFB and are characterized by the presence of early (blue) myelin degradation products in macrophages within the lesion (Figure 1g). In comparison to the NAWM astrocytes in A lesions already appear larger and more branched (Figure 1h). As expected for an active demyelination, there is no ongoing remyelination detectable, which is indicated by a total loss of OPCs in the lesion area (Figure 1i). The associated PLP additionally appears thinned out discernible by the pale pink background (Figure 1i).

In IA/ER (Figure 1j–l), the demyelinated area still appears in pink in LFB staining (Figure 1j) with macrophages containing late (pink) myelin degradation products, whereas the astrocytic network on an adjacent slide of the same lesion is already getting more dense (Figure 1k). The glial reaction coexists with marked ongoing remyelination. The highest number of OPCs can be found in IA/ER lesions, whereas the pale pink PLP immunoreactivity indicates substantial loss of PLP (Figure 1l).

Especially in later lesions stages, smooth lesion‐type transitions within one large lesion are most common in this experimental model with one large lesion containing edges of different stages of demyelination and remyelination. The rectangle in Figure 1m–o represents an IA/ER area with macrophages appearing with pink myelin degradation products in LFB staining (Figure 1m), slightly branched, activated astrocytes (Figure 1n), and a very high number of OPCs (Figure 1o). The arrows in Figure 1m–o are pointing at the LR border represented in LFB as a pink rim (Figure 1m), in GFAP staining as a heavily branched astrocytic network (Figure 1n) and with a decrease in OPC occurrence (Figure 1o). The circle in Figure 1m–o indicates the SP area, embedded in a rim of IA/ER and LR lesions. In LFB, this area appears light blue (Figure 1m) and in GFAP staining a very dense network represents the glial scar (Figure 1n). The massive counterstain indicated by the high amount of blue nuclei is one of the typical signs—some astrocytes have more than one nucleus in this lesion state. The OPC distribution approximates to those found in the NAWM (Figure 1o) with PLP staining still pale. The histopathological changes found in spinal cords were in line with the appearance of lesions found in the brain. Figure 2 shows an example of a large SP in the cerebellar white matter (myelin pallor in LFB staining, Figure 2b,c,e,f) as compared with NAWM area in Figure 2a,d. As in spinal cord lesions, the SP in the cerebellar white matter represented at the same time a dense glial scar with heavily intertwined astrocytic processes (Figure 2g–l). The number of OPCs however is in the range of those found in NAWM areas (Figure 2m–r).

### 3.2 Quantitative assessment of astrogliosis, OPCs, and mature oligodendrocyte occurrence in rat spinal cords

GFAP immunoreactivity was assessed in a total of 540 spinal cord lesions; in 450 lesions, the number of OPCs was quantified and for evaluation of mature oligodendrocytes we used a selected set of 120 representative lesions. Table 1 lists the total number of assessed lesions and the corresponding number of rats.

**Figure 3:**
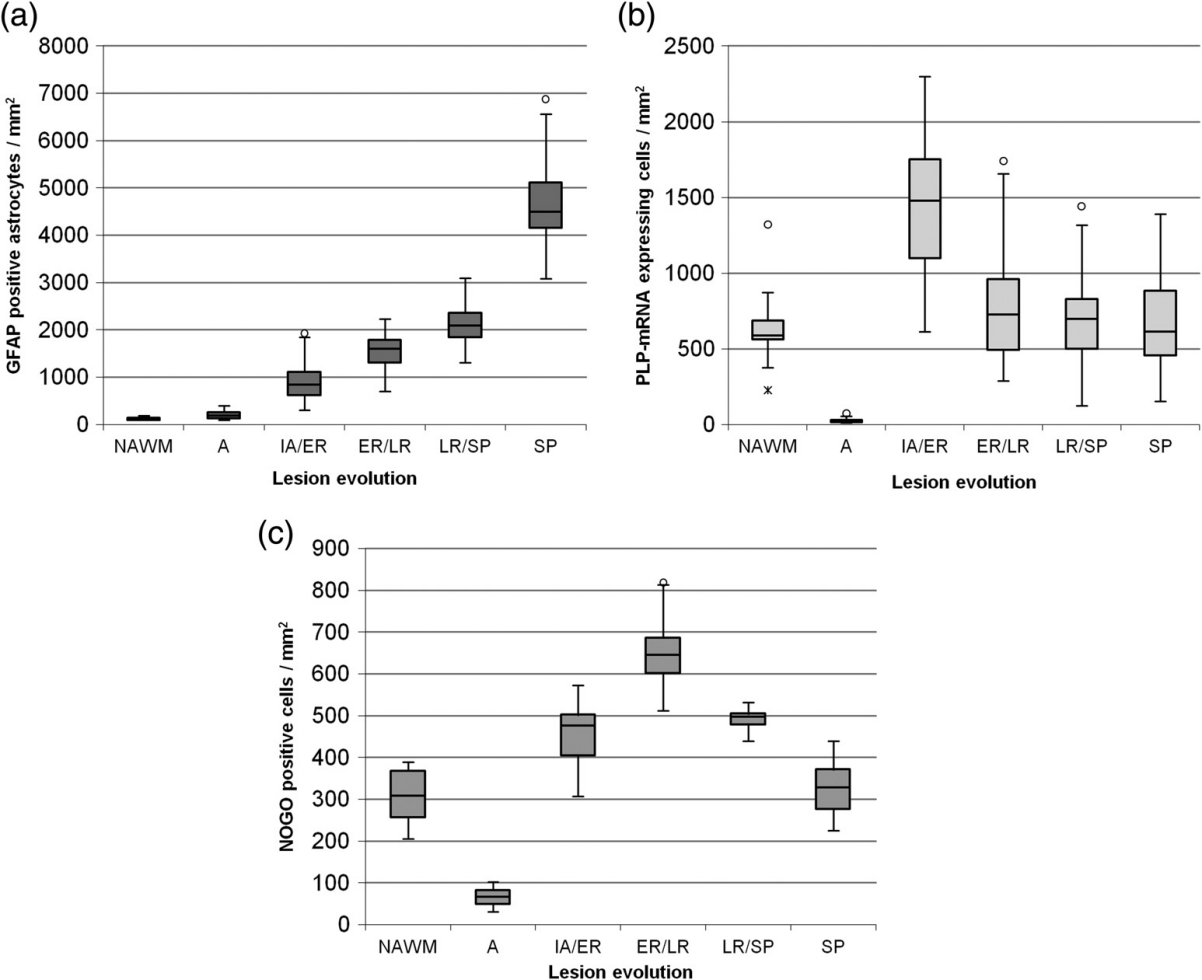
Statistical analyses of GFAP positive astrocytes, PLP mRNA expressing OPCs and NOGO positive mature oligodendrocytes. Astrogliosis as a marker of diseased tissue increases during lesion evolution and reaches its peak in SP representing all features known for a glial scar (a). In NAWM, only a few astrocytes are GFAP positive and a significant upregulation of GFAP expressing astrocytes is detectable in SP with almost all astrocytes expressing GFAP. All groups differ significantly from each other (*p* < .01). In total, 540 lesions were quantified for GFAP (out of 45 rats). The observation of OPCs during lesion evolution reveals in a peak of PLP mRNA expression in IA/ER (b). In A hardly any OPCs expressing PLP mRNA are detectable. PLP mRNA expression in fully remyelinated SP shows close resemblance to the expression in NAWM. ER/LR and LR/SP results are slightly over the range of detected OPCs in NAWM and SP. Only A and IA/ER lesions differ significantly from the other groups (*p* < .01). In total, 450 lesions were quantified for PLP (out of 45 rats). As expected the distribution of NOGO‐positive cells (mature oligodendrocytes) follow the course of OPCs with a slight delay. During A mature oligodendrocytes are comparably low. There is an increase in mature oligodendrocytes detectable during early remyelination indicating a successful differentiation of OPCs. Mature oligodendrocytes are also detectable during late remyelination and their occurrence in SP is comparable with their appearance in NAWM. All groups differ significantly from each other (*p* < .01) with the exception of NAWM to SP (*p* = .541) and IA/ER to ER/LR (*p* = .086). In total, 120 lesions were quantified for NOGO (out of 15 rats)

The results of the quantitative evaluation of GFAP positive astrocytes, representing the development of the glial scar, PLP mRNA positive OPCs as markers of remyelination in the different lesion types and NOGO positive cells as marker for mature oligodendrocytes of rat spinal cords are shown in box plots in Figure 3a–c, respectively. The corresponding number with median and percentiles are given in the Supporting Information Table S2.

With ongoing lesion evolution, the number of GFAP expressing astrocytes increased steadily from a median of 200 cells/mm^2^ in A lesions to SP with a peak of 4,500 cells/mm^2^, representing all features of a glial scar. All lesion types differed significantly from each other and NAWM in respect to the detected number of GFAP positive astrocytes (*p* = .000). The quantitative assessment of the glial scar formation can be followed in Figure 3a.

Active demyelination was associated with a loss of remyelinating and protecting cells indicated by a sharp drop of OPCs as compared with NAWM (median of 587 cells/mm^2^) to A (with a median of 20 cells/mm^2^). At the beginning of remyelination, there was an increase of OPCs detectable with a median of 1,479 cells/mm^2^ (*p* = .000). The median of detected PLP mRNA expressing OPCs in ER/LR, LR/SP, and SP was between 614 and 750 cells/mm^2^, which is close to the number of OPCs detectable in NAWM (*p*‐values NAWM to ER/LR = 0.157, NAWM to LR/SP = 0.185, and NAWM to SP = 0.893). The number of PLP mRNA expressing OPCs in ER/LR was slightly higher than in LR/SP (*p* = .223) and a decrease from ER/LR to SP (*p* = .175) could be observed, but these differences did not reach statistical significance. The quantitative evaluation of PLP mRNA expression of each lesion type is represented in a box plot in Figure 3b.

In addition to the loss of OPCs during active demyelination also a drop of mature oligodendrocytes could be seen from NAWM (median of 308 cells/mm^2^) to A (median of 67 cells/mm^2^; *p* = .000). During early remyelination, there was also an increase in mature oligodendrocytes detectable (median 476 cells/mm^2^). Most mature oligodendrocytes were found during early to late remyelination with a median of 640 cells/mm^2^. The number of cells decreased again during late remyelination to a number comparable to early remyelination (median 497 cells/mm^2^). In fully remyelinated SPs, the number of mature oligodendrocytes was comparable to that in NAWM with a median of 328 cells/mm^2^. The quantitative evaluation of NOGO‐IHC during lesion evolution is shown in a box plot in Figure 3c.

### 3.3 Investigation and quantitative assessment of astrocyte‐derived factors in rat spinal cords

**Figure 4:**
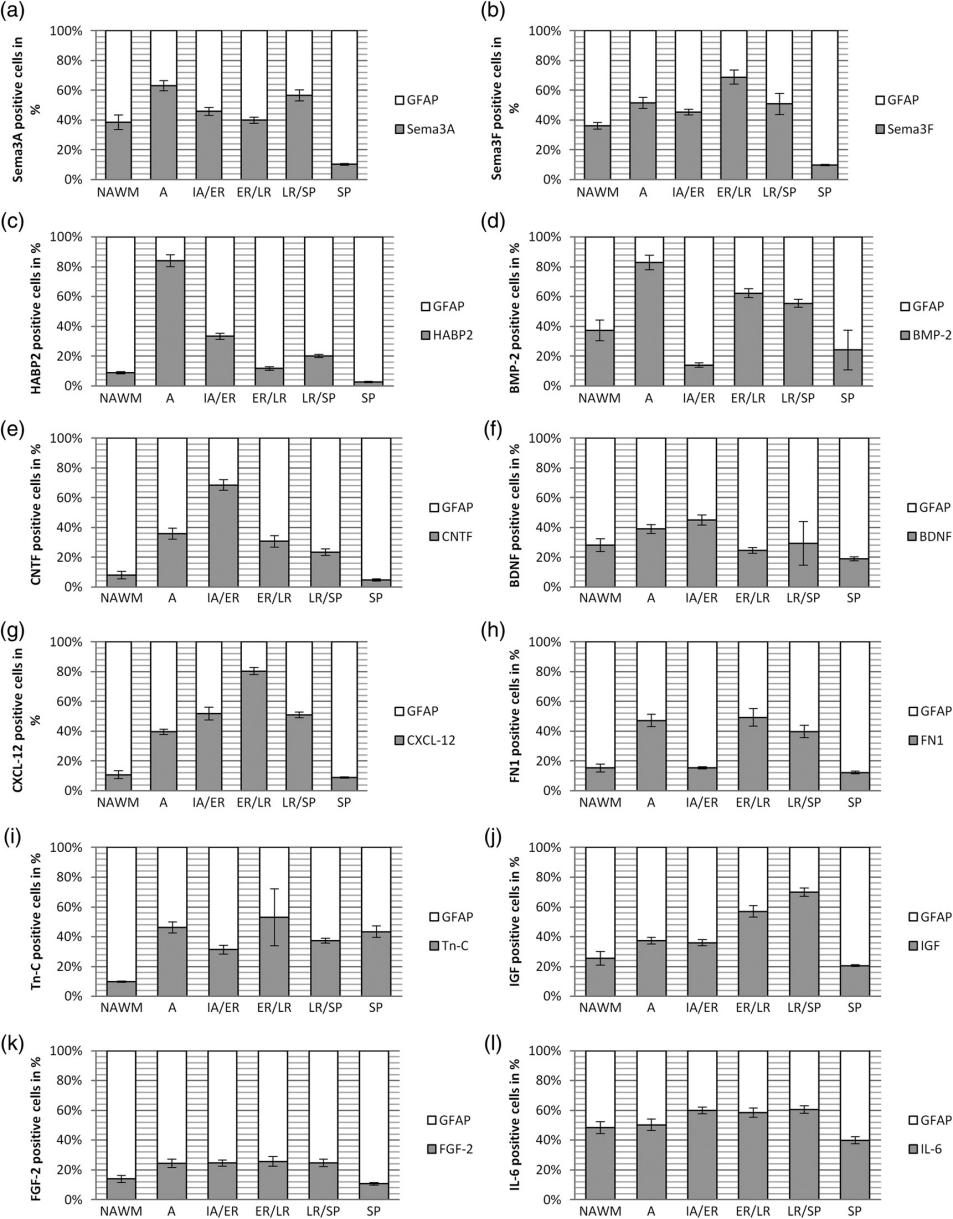
Percentage scale of GFAP positive astrocytes and astrocyte‐derived factors on selected spinal cord lesions. In each lesion type the mean of all astrocytes represents the 100% scale and the corresponding astrocytes expressing factors involved in OPC regulation are given in the appropriate ratios (for each factor n = 20 selected lesions per type). Error bars represent 95% CI. The guidance molecule Sema3A (a) peaked in A with more than 60% of active astrocytes expressing this factor and additionally showed a high occurrence in LR/SP with approximately 55%. In A, the expression of Sema3F (b) is higher than in IA/ER and peaks again in ER/LR. HABP2 (c) is highly present during A with more than 80% of all astrocytes expressing this factor. It decreases again to 5–30% during remyelination. BMP‐2 (d) is comparably high during A decreases in IA/ER and increases again during later remyelination steps. CNTF expression (e) increases during lesion evolution with a peak of almost 70% CNTF positive astrocytes in IA/ER and decreases again to approximately 5% in SP. BDNF (f) expression increases from NAWM to IA/ER, decreases in ER/LR, increases again in LR/SP and reaches its minimum in SP. CXCL‐12 (g) increases during lesion evolution, peaks in ER/LR with 80% of active astrocytes expressing this factor and decreases again from LR/SP to SP. Around 50% of all astrocytes are FN1 positive during A and late remyelination steps (h). Tn‐C is upregulated during A and ER/LR (i). The expression of IGF (j) rises from NAWM to LR/SP with a slight increase in A and falls rapidly from LR/SP to SP. FGF‐2 (k) showed only a slight increase from NAWM to A and decreases from LR/SP to SP. IL‐6 (l) shows a more uniform course with a slight increase of IL‐6 expression during remyelination process with more than 50% of all active astrocytes expressing IL‐6

**Figure 5:**
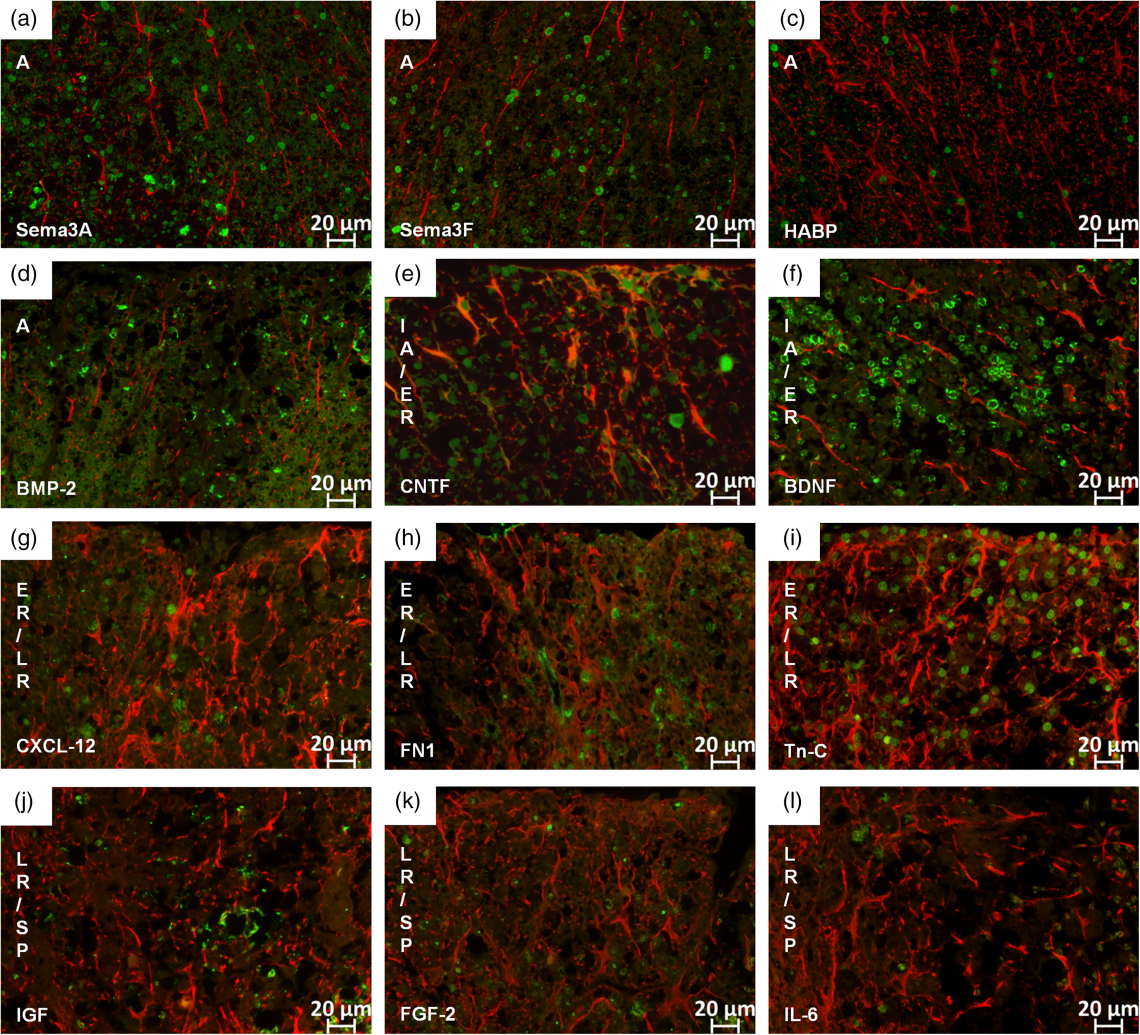
IF double staining for GFAP and selected astrocyte‐derived factors. In all figures GFAP appears in red and the respective factor in green. All factors are mainly present in the nucleus of the astrocytes. As Sema3A (a), Sema3F (b), HABP2 (c), and BMP‐2 (d) are mainly present during active demyelination those lesions are represented in the respective figure (a–d). Astrocytes are here in an early reactive state with mainly single nuclei and no branching. Factors mainly present during IA/ER are CNTF (e) and BDNF (f); astrocytes are enlarged and begin to branch at this stage. The factors CXCL‐12 (g), FN1 (h) and Tn‐C (i) are mainly present during ER/LR. During LR/SP we could detect more IGF (j) signal than FGF‐2 (k) and IL‐6 (l). (magnification: (a–l) 400×) [Color figure can be viewed at wileyonlinelibrary.com]

To further investigate the role of astrocytes and the plaque milieu in the different lesion types, we traced a set of astrocyte‐derived factors known for their involvement during lesion evolution and remyelination via IHC double staining of GFAP and the respective factor. For displaying the corresponding ratio of astrocytes expressing the respective factors, we chose a percentage scale. The selected set of astrocytic factors was quantitatively assessed in all different lesion types on representative slides (*n* = 20 per factor and lesion type). As already mentioned earlier, we encountered all investigated factors in all lesion types with different patterns of distribution (Figure 4). Some factors exhibited only small changes during lesion evolution; others underwent a remarkable change in expression pattern along the lesion evolution and the ongoing remyelination process. We arranged the factors according to their peak occurrence during lesion evolution and discuss the results systematically over the full course of lesion evolution. Figure 5 gives an overview of the appropriate lesions fitting to our statistical results.

During active demyelination, we found Sema3a, Sema3F, HABP2 and BMP‐2 to be highly up‐regulated (Figure 4a–d; Figure 5a–d). In this stage recruitment of OPCs already starts. We could detect a higher expression of Sema3A with 63% in this lesion type whereas only about 50% of astrocytes where expressing Sema3F. Interestingly, Sema3A was also very present in LR/SP (57%) and Sema3F in ER/LR (69%). HABP2 was highly expressed in active lesions by up to 80% of all detected astrocytes and BMP‐2 was very present during active demyelination with almost 85% of all astrocytes being BMP‐2 positive. BMP‐2 was also very highly expressed during LR.

For successful ER migration of OPCs to inactive lesions is necessary. Important factors highly expressed during this step in our sample were CNTF and BDNF (Figures 4e,f and 5e,f). CNTF peaked during ER with about 70% of all astrocytes expressing it and then decreased again during later phases of remyelination. BDNF only slightly increased during ER and was also present during active demyelination.

It is important for OPCs to be able to survive and differentiate in the lesion milieu during remyelination. In ER/LR lesions, we found CXCL‐12, FN1 and Tn‐C to be highly up‐regulated (Figures 4g–i and 5g–i). CXCL‐12 increased during remyelination to a maximum of 80% in ER/LR. Upon completion of remyelination their number decreased to 8% in the SP. More than 40% of all astrocytes were expressing FN1 during ER/LR but this factor was also comparable high during active demyelination and LR/SP lesions. Tn‐C increased during ER/LR up to almost 60% and was also high during active demyelination and LR/SP.

During LR/SP, we found IGF to be highly expressed with almost 70% of all astrocytes being positive (Figures 4j and 5j) followed by a remarkable decrease to 21% upon reaching the SP.

FGF‐2 and IL‐6 showed both a rather uniform course with only small increases and a rather constant level of expression over the entire lesion evolution (Figures 4k,l and 5k,l).

We conducted a correlation analysis with all factors and OPC density. The calculated Sperman‐Rho correlation coefficient is shown in Table 2. There was a strong correlation between OPC density and Sema3A, Sema3F, CNTF, CXCL‐12, FN1, and IGF, respectively. All correlation analyses reached significance with only one exception (HABP2; *p* = .160).

**Table 2:** Sperman‐rho correlation results of astrocytic factors

Correlation of OPC density and	Correlation coefficient	Significance (two‐tailed)
Sema3A*	0.622	.000
Sema3F*	0.772	.000
HABP2	0.142	.160
BMP2*	0.406	.000
CNTF*	0.750	.000
BDNF*	0.262	.009
CXCL‐12*	0.833	.000
FN1*	0.577	.000
Tn‐C*	0.338	.001
IGF*	0.589	.000
FGF*	0.498	.000
IL‐6*	0.339	.001

A *p*‐value of .01 was considered as significant and is marked with a*. Strong correlations are marked with an underline.

### 3.4 The switch of astrocytic phenotypes during lesion evolution

Neuroinflammation induces two different types of reactive astrocytes that were termed as A1 and A2 according to Liddelow et al., 2017. A1 are described as regulating classical complement cascade genes and postulated as harmful. In contrast, A2 astrocytes regulate many neurotrophic factors and are described as being protective (Liddelow et al., 2017).

**Figure 6:**
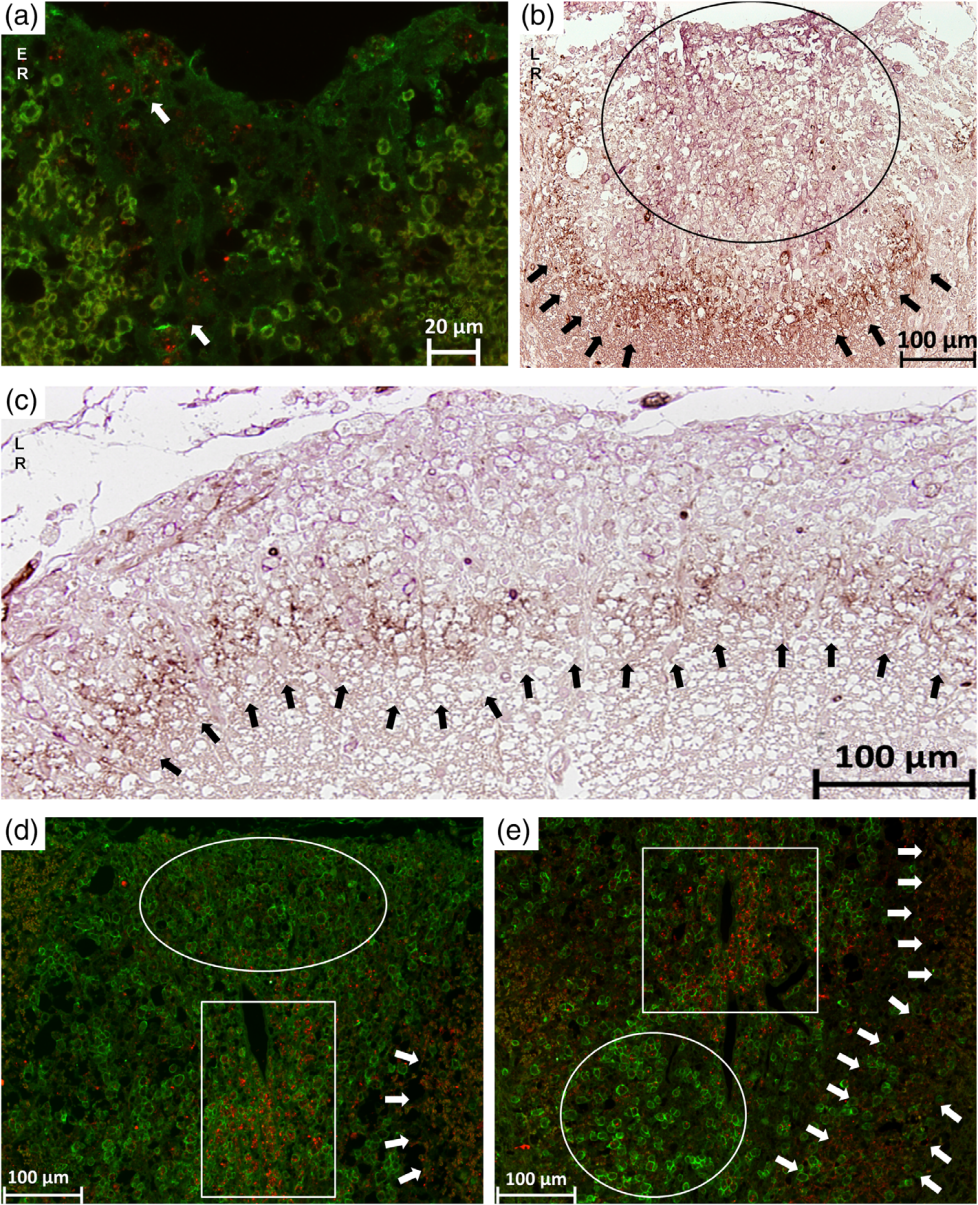
The switch of astrocytic phenotypes A1 and A2 during lesion evolution. In all IF stainings, the A1 phenotype (C3d) appears in red and the A2 phenotype (S100A10) in green. In all IHC stainings, the A1 phenotype appears in brown and the A2 phenotype in purple. In (a) a early remyelinating lesion is shown on a IF stained slide where the A2 phenotype is mainly present and the A1 phenotype is spread over the lesion in a granular‐like pattern indicated by the arrows. In (b) a IHC of a late remyelinating lesion is marked by the circle where the A2 phenotype (purple) is mainly present. The arrows point at the lesion border where the A1 phenotype (brown) accumulates. This remarkable border is also shown in another late remyelination lesion in (c) indicated by the arrows. In (d) and (e), IF stainings are shown with different lesion types side‐by‐side. The circles show SPs where the A2 phenotype (green) is mainly present. In the rectangles early remyelinating areas are marked with the A1 phenotype (red) spread over in a granular‐like pattern. The arrows show the border of A1 phenotype, which was detectable during later remyelination steps in our sample [Color figure can be viewed at wileyonlinelibrary.com]

In NAWM, both A1 and A2 phenotypes were detectable with a twofold higher number of A2 astrocytes (83 ± 9.8) than A1 (40 ± 9.4). In active lesions more than twice as much astrocytes were carrying the A1 phenotype (195 ± 13.6) and only a moderate number carrying A2 (90 ± 8.6). Interestingly, we found almost all astrocytes being A2 (green) in IA/ER lesions (604 ± 30.0) and the marker of A1 (red) spread over the whole lesion in a granular manner (Figure 6a). From ER/LR lesions to LR/SP lesions almost all astrocytes showed the A2 (purple) phenotype within the lesion with a surrounding small rim of A1 (brown) astrocytes bordering the lesion (Figure 6b,c). In fully remyelinated SP, the majority of astrocytes showed the A2 (green) phenotype (4,625 ± 448.1) and only a few astrocytes exhibited A1 (red) characteristics (22 ± 10.1; the circle in Figure 6d,e).

## 4 DISCUSSION

Remyelination of the CNS after a demyelinating event proceeds in distinct stages. OPCs are recruited into demyelinated areas and differentiate into myelinating oligodendrocytes to form new myelin sheaths (Horner & Gage, 2000; Kotter, Stadelmann, & Hartung, 2011). PLP is one of the major proteins in compact myelin and its expression occurs very early in development in a subset of progenitor cells. PLP mRNA therefore is a valid marker to trace the OPC infiltration and the process of remyelination (Harlow, Saul, Culp, Vesely, & Macklin, 2014; Lindner et al., 2008). The basic requirement for remyelination is the presence of axons and OPCs as well as the correct interaction of extrinsic signaling pathways. The migration of OPCs is controlled by secreted molecules such as growth factors, guidance molecules, and chemokines, many of them expressed by astrocytes. Depending on their activation state, astrocytes can exert both remyelination facilitating and potent suppressive effects on OPCs (Hagemeier, Brück, & Kuhlmann, 2012; Itoh et al., 2018; Nair et al., 2008; Sofroniew & Vinters, 2010), but especially the formation of a dense glial scar is often believed to act as an impenetrable mechanical barrier for any remyelination efforts.

In this experimental study, we show that in our sample of MOG EAE in DA rats, the astrocytic reaction increases over the time course of lesion evolution from active demyelination to successfully completed remyelination. The SP, characterized by newly formed, slightly thinner myelin sheaths represented at the same time a dense glial scar in GFAP staining, which according to the current literature should prevent OPCs from entering the tissue and counteract remyelination (Nair et al., 2008). The number of OPCs in this lesion area however equaled the range of OPCs found in the NAWM, indicating that OPCs, on the contrary, must be able to migrate and proliferate very well even in this environment. This is furthermore supported by our investigation of mature oligodendrocytes in the same sample—an equal number of oligodendrocytes was found in NAWM and SP.

By tracing OPCs via ISH for PLP mRNA as well as mature oligodendrocytes over the full lesion course, we found that after a nearly complete loss of OPCs during active demyelination they are recruited back again once the lesion becomes inactive. As anticipated, the highest amounts of PLP mRNA, representing increased remyelinating activity of OPCs, were found in IA/ER lesions. Upon successfully completed remyelination, PLP mRNA decreased again. The remarkable decrease in PLP mRNA expressing OPCs from IA/ER to later remyelination stages is explainable by the maturation of OPCs to myelinating oligodendrocytes. Accordingly, the highest number of mature oligodendrocytes was detectable in ER/LR. The SP exhibited an OPC and mature oligodendrocyte number as seen in the NAWM despite showing features of a dense glial scarring. We concluded, therefore, that extensive glial reaction and even scar formation does not per se hinder migration and survival of OPCs but, on the contrary, might even allow and support remyelination in the presence of the right mediators facilitating repair at the right lesion stage.

To test this hypothesis, we investigated the expression of several astrocyte‐derived factors with assumed influence on remyelination over the full course of lesion evolution to gain more insight into the plaque environment at every lesion stage. Based on the available literature, we concentrated on a subset of exemplary astrocytic factors and quantitatively assessed the percentage of astrocytes in the lesions expressing the respective factor.

The first step for a successful repair is the appropriate recruitment of OPCs into the lesion itself. There are several factors known to be necessary for OPC guidance. We found factors with different properties and functions at this stage. Sema3A and Sema3F are on the one hand known in the literature as guidance molecules for axons and OPCs in the developing nervous system and are also upregulated in MS brains in glial cells and neurons in the vicinity of active demyelinating lesions. Especially the expression of Sema3F is believed to serve as a recruitment signal for OPCs into the lesion (Williams, de Wit, & Ghosh, 2010). In contrast, both Semaphorines are also discussed as reversible inhibitors of OPC differentiation (Syed et al., 2011). In our sample, we detect high levels of both Sema3A and Sema3F expression in all lesion types with a peak of Sema3A in A and LR and of Sema3F in ER/LR, obviously representing sequential recruitment signals on OPCs during remyelination, at the same time maybe preventing too early differentiation of OPCs. We hypothesize that besides a guidance function also the inhibition of OPC differentiation is necessary at this lesion stage. As OPCs have to be recruited first, a too early differentiation might be detrimental for migration to the particular lesion.

The second factor of importance at this lesion stage synthesized by astrocytes and analyzed in our work was hyaluronan (Back et al., 2005). Hyaluronan deposits accumulate in demyelinated lesions in MS patients and in mice with EAE and are discussed to inhibit OPC maturation (Sloane et al., 2010). In our sample, we found hyaluronan to be present in 80% of astrocytes during active demyelination followed by a decrease during remyelination. This finding is in line with the assumption that the blocking effect of hyaluronan on OPCs seems to be reversible and controlled by enzymatic degradation (Sloane et al., 2010) and supports our hypothesis that blocking of maturation is necessary during guidance and migration steps.

Another factor described during the stage of OPC guidance is BMP‐2. It is thought to promote astrocyte reactivity and stimulate glial scar formation. BMPs in general are discussed to decrease proliferation of precursors, increase apoptosis, and increase expression of GFAP. Increase in BMP‐2 is discussed to be an early signal stimulating glial scar formation after demyelinating injuries (Fuller et al., 2007). It is furthermore described to inhibit the production of oligodendrocytes in the healthy CNS. As BMPs are expressed in demyelinating lesions in human brains, it is even described to be a potential therapeutic target to enhance repair (Sabo, Aumann, Merlo, Kilpatrick, & Cate, 2011). In our sample, we found BMP‐2 mainly during active demyelination and late remyelination, but it was downregulated during early remyelination. The rather negative view of BMP‐2 regarding its impact on remyelination in the literature most likely correlates with the—most often—negative connotation of the glial scar. When describing BMP‐2 as a promotor of astrogliosis, it is often described as inhibitor of remyelination. We suggest here a rather supportive role of the glial scar than a detrimental one, which may also changes the view of BMP‐2.

The next mandatory step for successful remyelination is the migration of OPCs into the lesion area and the associated start of repair. We therefore followed the two factors CNTF and BDNF known for these properties in our sample. CNTF was shown to promote survival, migration, and differentiation of OPCs into mature myelin forming cells (Modi, Sendtner, & Pahan, 2013). Furthermore, CNTF was additionally shown to promote the survival of neurons and to protect mature neurons following CNS injury (Dallner, Woods, Deller, Kirsch, & Hofmann, 2002; Linker et al., 2002; Yokota et al., 2005). In our sample, CNTF positive astrocytes reached a peak in IA/ER of 70%; in A and ER/LR the percentage was still over 30%. The parallel peak of CNTF expression and accumulation of OPC suggests a potent communication between this factor and this cell type. Presumably the high expression of CNTF allowed better survival of OPCs in our experimental animals than in MS brains, where remyelination often fails. Induction of CNTF signaling has already been discussed as a potential therapeutic target in MS patients (Dutta et al., 2007).

Another astrocyte‐derived factor mainly thought to be involved in promoting migration of OPCs is BDNF. It is a member of the nerve growth factor (NGF) neurotrophin family and known as mediator of adult neurogenesis (Watts, Mcconkey, Anderson, & Caldwell, 2005). Furthermore, it was shown to prevent axonal and neuronal damage after various pathological insults to the brain (Gravel, Götz, Lorrain, & Sendtner, 1997; Weibel, Kreutzberg, & Schwab, 1995). In MS brain specimen, it is primarily found in immune cells and reactive astrocytes with the highest number of BDNF positive cells being found at the actively demyelinating edge early in the development of a MS lesion, most likely to protect nerve cell processes at risk of bystander damage (Stadelmann et al., 2002). In line with this assumption, in our experimental sample, we could detect an increase in BDNF positive astrocytes from A to IA/ER and a decrease during late remyelination.

The next mandatory step to a successfully remyelinated lesion is the differentiation of OPCs to mature oligodendrocytes. CXCL‐12, FN1, and Tn‐C are exemplary factors supporting this critical step. Chemokines are important keyplayers in the regulation of OPCs and interact with chemokine‐receptors on the OPC cell‐surface. In the normal CNS CXCL‐12 is constitutively expressed on endothelial cells and a small number of astrocytes, while in MS CXCL‐12 levels were shown to be high on astrocytes in both active lesions and at the edges of silent lesions where repair processes were taking place (Calderon et al., 2006). In line with this, the percentage of CXCL‐12 expressing astrocytes in our sample increased steadily from A to ER/LR lesions, where it reached its maximum. Upon completion of remyelination, values dropped again and only a few single astrocytes still expressed CXCL12 in shadow plaques, similar to the NAWM. This expression pattern is comparable with the time course of our OPC observations but with a slight delay. During active demyelination, in the absence of OPCs, the factor is already detectable in our sample, probably in preparation for OPC migration in IA/ER. CXCL‐12 might also help during differentiation of OPCs explaining the very high level in ER/LR. In SP, however, the expression of CXCL‐12 gets back to a level similar to that observed in the NAWM.

In chronically demyelinated MS lesions, FN1 expression is found in the form of aggregates suggested to be resistant to degradation. Those aggregates are described in the literature to contribute to remyelination failure by inhibiting OPC differentiation (Stoffels et al., 2013). In our work, we found FN1 to be upregulated (present in around 50% of all detected astrocytes) during active demyelination as well as in later stages of remyelination. During active demyelination, it might again be necessary to prevent OPCs from too early differentiation. Whether FN1 could indeed be detrimental or not during later remyelination has to be investigated further.

Tn‐C is described in the pathogenesis of CNS autoimmunity and various pro‐inflammatory mediators have been shown to induce expression of Tn‐C (Momčilović et al., 2017). In line with this observation, we found a Tn‐C peak with 50% of all astrocytes expressing this factor during active demyelination. In addition, Tn‐C production by reactive astrocytes is suggested to result in glial scar formation impeding remyelination and axonal repair in MS lesions (Gutowski, Newcombe, & Cuzner, 1999). In our work, Tn‐C was also present during remyelination, which may support the presence of astroglial reaction. Similar to BMP‐2, the view on this factor depends on the perception of the impact of the glial scar. However, the role of Tn‐C is indeed much more complex and our investigation can only reflect a small part of it. Tn‐C is, for example, further described to disturb the blood‐brain barrier in an experimental mouse model and it is also upregulated and detectable in MS sera and lesions (Gutowski et al., 1999; Harada et al., 2015). In addition, it is described that Tn‐C deficiency protects against EAE (Momčilović et al., 2017).

Finally, OPCs must be able to survive and proliferate in the lesion milieu. IGF is one factor that is discussed as supporting this function. In animal models of MS, an astrocytic IGF upregulation in demyelinating lesions was found (Hinks & Franklin, 1999) and in the absence of IGF signaling remyelination does not adequately occur as OPCs do not proliferate or survive (Mason, Xuan, Dragatsis, Efstratiadis, & Goldman, 2003). In our sample, numbers of IGF expressing astrocytes increased steadily from A to LR lesions where they reached their maximum. In SP, IGF expression was similar to the pattern found in NAWM. Comparing these observations to our OPC findings, we suggest a role of IGF in OPC survival, maturation, and myelin formation.

The two factors FGF‐2 and IL‐6 in our sample set were present in a rather uniform manner during lesion evolution and we could not detect any clear peaks. FGF‐2 is a mediator of adult neurogenesis and described in the literature to be involved in the recruitment of OPCs (Watts et al., 2005). Recent research showed that FGF‐2 is highly expressed by active astrocytes in the subventricular zone in the adult brain, enhances the recruitment of progenitor cells, and therefore facilitates myelin repair (Azin, Mirnajafi‐Zadeh, & Javan, 2014; Huang & Dreyfus, 2016). In experimental demyelination, FGF‐2 mRNA levels peaked at the initial stage of remyelination supporting a remyelination‐enhancing role (Messersmith, Murtie, Le, Frost, & Armstrong, 2000). Furthermore, in EAE FGF‐2 has been identified as a neuroprotective factor preventing and even treating this disease (Woodbury & Ikezu, 2014), whereas another animal study found, on the contrary, a demyelinating effect of continuously increased levels of FGF‐2 and an inhibitory effect on OPC differentiation (Zhou, Flint, Murtie, Le, & Armstrong, 2006). In line in our study, FGF‐2 was expressed on astrocytes in all lesion types but rather at uniformly low levels.

IL‐6 and CNTF are structurally and functionally related molecules and both belong to the family of neuropoietic cytokines (Schönrock, Gawlowski, & Brück, 2000). In MS brain specimens, IL‐6 was observed in only 10–17% of the astrocytes (Schönrock et al., 2000). In contrast to this finding, in our sample, we detected a constantly expression level of 40–60% of IL‐6 positive astrocytes over all lesion types.

When looking at the results of astrocytic factors, our findings suggest a very fine graduated and balanced system with different factors having to be present at particular time points to archive successful repair. It is often not possible to identify some factors as purely remyelination facilitating and some others as detrimental; rather the individual factors appear to have a distinct function at a distinct lesion stage. Recent work of Anderson et al. showed that preventing glial scar formation, attenuating scar forming astrocytes, or deleting chronic astrocyte scars in severe spinal cord injury lesion in mice failed to result in a regrowth of axons. They even found out that a loss of the glial scar prevents stimulated axon regrowth. They therefore concluded that the glial scar formation rather aids than prevents axon regeneration (Anderson et al., 2016).

In addition to the formation of a glial scar, the very complex role of astrocytes is further characterized by their ability to exhibit different phenotypes and thereby to broaden their operational area. Neuroinflammation induces two different types of reactive astrocytes that were named as “A1” and “A2”, respectively (Liddelow et al., 2017). A1 astrocytes are induced by classically activated neuroinflammatory microglia and were described as harmful by inhibiting OPC proliferation and differentiation, whereas A2 astrocytes were suggested to be protective. In line with that we found A1 astrocytes mainly present in active lesions and the A2 phenotype during remyelination. Interestingly, the distribution of the A1 phenotype also changed with the start of remyelination. During early remyelination, A1 was detectable in a granular pattern spreading over the lesion. During later remyelination, the A1 phenotype formed a ring surrounding the lesion, which might acts as a barrier, protecting adjacent healthy tissue.

## 5 CONCLUSION

From our data, we conclude that the formation of a glial scar after a demyelinating event does not per se hinder OPC survival, migration nor remyelination, presumably as long as a multitude of different cellular mediators are finely tuned in correct succession and dosage. This supports the new view of the glial scar not being a rigid border but rather provides a complex milieu of different factors influencing oligodendrocytes and presumably other cell types too. The sometimes contradicting findings from animal and cell culture studies however show how complex and poorly understood the underlying signaling mechanisms still are. Regional differences between astrocytes, their activation state and the time point after destruction could very likely determine their overall function pro‐ or contra remyelination. Furthermore, in MS patients as well as in EAE, there are different lesion types coexisting at each given point in time. As lesions are no rigid, strictly bordered structures the cellular milieu can also be influenced through neighboring lesions. This dichotomy in astrocyte function makes these cells an interesting, though challenging target for new therapeutic strategies. A limitation of our work comes from the fact that we investigated experimental material only and additional systematic studies with human autopsy material of MS patients are necessary to validate our results for the human situation.

## CONFLICTS OF INTEREST

The authors declare no conflicts of interest.

## Supporting information

Supplementary Figure S1 **Supplementary figure Validation of astrocytic marker GFAP, Nestin and Vimentin, co‐expression study of GLT1/GFAP and verification of myelin digestion products of macrophages.**
The validation of astrocytic markers is shown on IHC stainings (a‐i). Each row represents another astrocytic marker on adjacent lesions from active lesions to late remyelination. In our work we chose GFAP (a‐c) because of its best countability. The marker Nestin (d‐f) detects a lower number of astrocytes in comparison to GFAP. From (g) to (j) the same lesions are shown immunohistochemically stained for Vimentin. There is a close similarity to GFAP during A and IA/ER but during later remyelination steps astrocytes are more difficult to detect. In (j) we show a co‐expression of GLT1 and GFAP on a ER/LR lesion. GLT1 is shown in green and comparatively less expressed than GFAP here shown in red. The verification of myelin digestion products within macrophages is shown via IF stainings in (k) and (l). Macrophages (ED1) are represented in red and myelin (MOG) in green. In active lesions macrophages contain green myelin products (k) indicated by the arrows. In (l) an inactive lesion area is shown, where the myelin is already digested within the macrophages and therefore macrophages appear only in red, indicated by the arrows

Supplementary Table S1 The following antibodies have been used

Supplementary Table S2 Median and percentile of GFAP, PLP and NOGO quantification
