# Supplementary figures and images for: The formation of a glial scar does not prohibit remyelination in an animal model of multiple sclerosis
Source: Glia. 2018 Nov 28;67(3):467–81. doi: 10.1002/glia.23556 (PMC6588096; doi:10.1002/glia.23556)

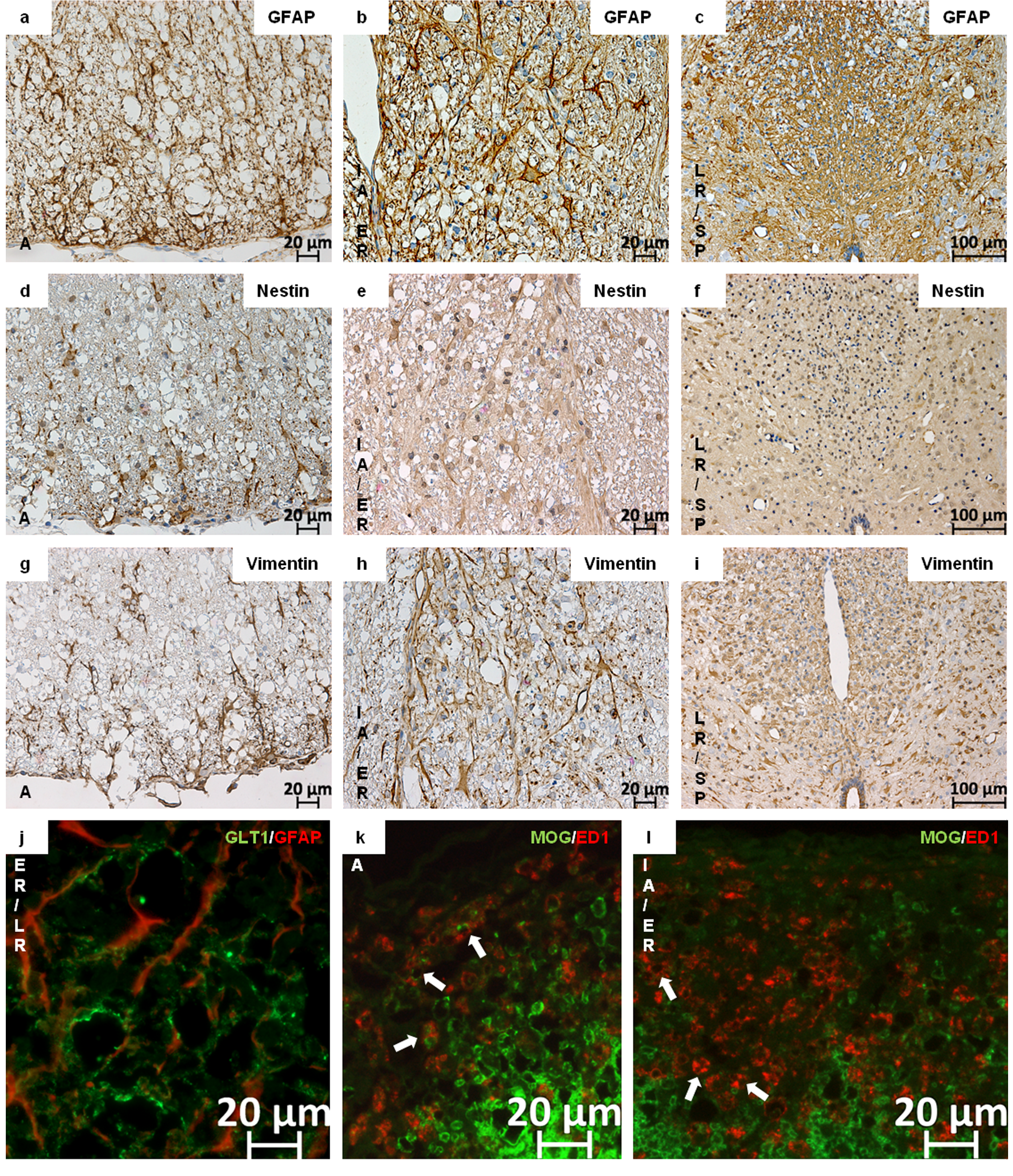

Supplement: Supplementary file 1 — Supplementary Figure S1 Supplementary figure Validation of astrocytic marker GFAP, Nestin and Vimentin, co‐expression study of GLT1/GFAP and verification of myelin digestion products of macrophages. The validation of astrocytic markers is shown on IHC stainings (a‐i). Each row represents another astrocytic marker on adjacent lesions from active lesions to late remyelination. In our work we chose GFAP (a‐c) because of its best countability. The marker Nestin (d‐f) detects a lower number of astrocytes in comparison to GFAP. From (g) to (j) the same lesions are shown immunohistochemically stained for Vimentin. There is a close similarity to GFAP during A and IA/ER but during later remyelination steps astrocytes are more difficult to detect. In (j) we show a co‐expression of GLT1 and GFAP on a ER/LR lesion. GLT1 is shown in green and comparatively less expressed than GFAP here shown in red. The verification of myelin digestion products within macrophages is shown via IF stainings in (k) and (l). Macrophages (ED1) are represented in red and myelin (MOG) in green. In active lesions macrophages contain green myelin products (k) indicated by the arrows. In (l) an inactive lesion area is shown, where the myelin is already digested within the macrophages and therefore macrophages appear only in red, indicated by the arrows [file GLIA-67-467-s001.tif]
