# Supplementary material for: The formation of a glial scar does not prohibit remyelination in an animal model of multiple sclerosis
Source: Glia. 2018 Nov 28;67(3):467–81. doi: 10.1002/glia.23556 (PMC6588096; doi:10.1002/glia.23556)
Supplement: Supplementary file 2 — Supplementary Table S1 The following antibodies have been used [file GLIA-67-467-s002.docx]

**Supplementary table 2:** Median and percentile of GFAP, PLP and NOGO quantification.

| **GFAP** | | | |
| --- | --- | --- | --- |
|  | **Median** | **25. Percentile** | **75 Percentile** |
| **NAWM** | 102.00 | 98.00 | 139.00 |
| **A** | 194.00 | 133.00 | 266.00 |
| **IA/ER** | 843.50 | 620.75 | 1120.25 |
| **ER/LR** | 1600.00 | 1302.25 | 1793.00 |
| **LR/SP** | 2088.50 | 1838.00 | 2366.75 |
| **SP** | 4498.00 | 4133.50 | 5131.50 |
| **PLP** | | | |
|  | **Median** | **25. Percentile** | **75 Percentile** |
| **NAWM** | 587.00 | 563.00 | 701.50 |
| **A** | 20.00 | 13.50 | 32.50 |
| **IA/ER** | 1479.00 | 1083.25 | 1757.50 |
| **ER/LR** | 726.50 | 491.00 | 964.50 |
| **LR/SP** | 696.00 | 491.50 | 854.50 |
| **SP** | 614.00 | 450.00 | 890.00 |
| **NOGO** | | | |
|  | **Median** | **25. Percentile** | **75 Percentile** |
| **NAWM** | 307.50 | 256.00 | 368.00 |
| **A** | 66.50 | 43.50 | 82.00 |
| **IA/ER** | 476.00 | 394.00 | 502.00 |
| **ER/LR** | 645.50 | 575.75 | 686.00 |
| **LR/SP** | 496.50 | 473.50 | 509.50 |
| **SP** | 325.00 | 276.00 | 376.25 |
